# Constructing a Consensus on Language Evolution? Convergences and Differences Between Biolinguistic and Usage-Based Approaches

**DOI:** 10.3389/fpsyg.2019.02537

**Published:** 2019-11-14

**Authors:** Michael Pleyer, Stefan Hartmann

**Affiliations:** ^1^English Department, University of Koblenz-Landau, Koblenz, Germany; ^2^German Department, Chair of German Linguistics, University of Bamberg, Bamberg, Germany

**Keywords:** usage-based linguistics, construction grammar, biolinguistics, cognitive linguistics, evolutionary linguistics

## Abstract

Two of the main theoretical approaches to the evolution of language are biolinguistics and usage-based approaches. Both are often conceptualized as belonging to seemingly irreconcilable “camps.” Biolinguistic approaches assume that the ability to acquire language is based on a language-specific genetic foundation. Usage-based approaches, on the other hand, stress the importance of domain-general cognitive capacities, social cognition, and interaction. However, there have been a number of recent developments in both paradigms which suggest that biolinguistic and usage-based approaches are actually moving closer together. For example, theoretical advancements such as evo-devo and complex adaptive system theory have gained traction in the language sciences, leading to changed conceptions of issues like the relative influence of “nature” and “nurture.” In this paper, we outline points of convergence between current minimalist biolinguistic and usage-based approaches regarding four contentious issues: (1) modularity and domain specificity; (2) innateness and development; (3) cultural and biological evolution; and (4) knowledge of language and its description. We show that across both paradigms, researchers have come to increasingly embrace more complex views of these issues. They also have come to appreciate the view that biological and cultural evolution are closely intertwined, which lead to an increased amount of common ground between minimalist biolinguistics and usage-based approaches.

## Introduction

As Jackendoff (2010) famously stated, “[y]our theory of language evolution depends on your theory of language.” However, the converse is also true: Looking at language “in the light of evolution” (Dobzhansky, 1973; Hurford, 2007, 2012) can inform theories of language. For instance, Johnson (2017, p. 171) points out that “Chomsky’s Minimalist Program is largely motivated by the challenge of explaining the evolution of language.” For usage-based and emergentist approaches, the evolutionary dimension is also central to their view of language. In these approaches, language is seen as a complex adaptive system emerging out of the multifactorial and non-linear interactions of factors on ontogenetic, cultural, and evolutionary timescales (e.g., Beckner et al., 2009; Steels, 2011; Kirby, 2017).

However, minimalist biolinguistics and usage-based approaches have traditionally adopted quite opposing views on central issues such as modularity, domain specificity vs. domain generality, as well as innateness and development. As is well-known, these different views have been part of a long-standing controversy in linguistics (see e.g., Harris, 1993) that has recently been fueled by a number of publications (e.g., Evans, 2014; Dąbrowska, 2015; Adger, 2015a,b, among many others). Naturally, this divide also has repercussions for the field of language evolution research: For example, Johansson (2014) deplores a deep divide “between Chomskyan biolinguistics and everybody else” and speaks of a “Kuhnian incommensurability problem,” alluding to the mutually incompatible ways of viewing the world that different schools of thought in science tend to develop, which Kuhn (1970) sees as characteristic of scientific revolutions.

However, the views on these complex issues have not remained static within these approaches but have evolved considerably, especially in recent years. They also continue to play an important role in research on the evolution of language, as indicated, for example, in the recent collections of articles by Fitch (2017), Ferretti et al. (2018), and Petkov and Marslen-Wilson (2018). Intriguingly, in many ways both minimalist biolinguistics, on the one hand, and usage-based and emergentist approaches, on the other, have moved closer together in their conceptualizations of these long-standing issues. It can be argued that the convergent conceptual evolution seen in both approaches is in large part influenced by the fact that both biolinguistics and usage-based approaches have become increasingly interdisciplinary and empirically minded in their outlook. As we argue in this paper, by integrating perspectives and results from the cognitive and biological sciences such as evolutionary-developmental biology (evo-devo) and complex adaptive systems, both fields are in fact moving toward convergent conceptualizations on a number of key issues.

In this paper, we discuss these potential points of convergence, but we also show where there is still considerable dissent between the different paradigms. We would like to stress at the outset that the notions of “biolinguistics” and “usage-based approaches” that we are going to use in this paper are of course gross idealizations. Needless to say, the theoretical approaches subsumed under these umbrellas are wildly different. Nevertheless, traditionally these two approaches have been characterized by their divergences on issues of modularity, domain specificity, innateness, and development that we would like to highlight in this paper. While biolinguistic and usage-based approaches obviously provide very different answers to these questions, the potential convergence that we would like to highlight in this article crucially relies on empirical findings that have recently become available.

In many respects, biolinguistics and usage-based approaches have very different goals and different perspectives on what “knowledge” of language entails, how it is represented, how it is acquired, and how it emerged both culturally/historically and evolutionarily. We believe that many of these differences are not going to be resolved anywhere in the near future – and in terms of different goals and interests, resolution might not even be necessary. However, one of the main points of our paper is that regardless of seemingly “irreconcilable” differences, it is worth pointing out what biolinguistics and usage-based approaches have in common and where we see potential and opportunity for even further convergences and overlap in the future.

We will start by characterizing in broad strokes the two frameworks, *Usage-Based Approaches,* on the one hand, and *Biolinguistics,* on the other, to give a general conceptual map for comparison. Then, we will turn toward *Convergence and Divergence* between these approaches. First, we will focus on *Modularity and Domain Specificity*. Following this, we will discuss emerging trends in the way *Innateness and Development* are conceptualized in biolinguistics and usage-based approaches. We will then consider how these developments influence the way these approaches investigate the *Biological and Cultural Evolution* of language as well as their interrelation. Finally, we will deal with a number of theoretical and methodological differences between usage-based approaches and biolinguistics regarding *Knowledge of Language and Its Description*, which still set the two approaches apart quite clearly. We will end with a concluding summary of the potential for convergence and remaining divergences and with a call for further cross-fertilization and dialogue.

## The Frameworks

### Usage-Based Approaches

Under the heading “usage-based approaches,” we subsume a variety of frameworks that share a number of important assumptions. These approaches include but are not limited to Cognitive Linguistics (e.g., Geeraerts and Cuyckens, 2007; Dąbrowska and Divjak, 2015; Dancygier, 2017), Construction Grammar (e.g., Goldberg, 1995, 2003; Hoffmann and Trousdale, 2013; Diessel, 2015), and Functional-Cognitive Approaches (Butler and Gonzálvez-García, 2014). Usage-based approaches can be counted as belonging to the general approach of emergentism (e.g., MacWhinney and O’Grady, 2015), and we will often use the terms “usage-based” and “emergentist approaches” interchangeably.

First of all, usage-based approaches assign a key role to language usage. As Tomasello (2009, p. 69) puts it, “meaning is use – structure emerges from use.” This means that in these approaches, linguistic knowledge, and knowledge of constructions, proceeds *via* the abstraction and schematization of actual language use in context, yielding fixed chunks as well as more abstract linguistic patterns that become cognitively entrenched. This also means that, secondly, they tend to reject the notion of an innate Universal Grammar. Third, and also related to this, the rejection of a specific “language organ” of any kind usually goes in tandem with the assumption that cognition in general is “continuous” and distributed rather than modular (see Spivey, 2007). That is, language is thought to be based on general cognitive mechanisms (domain-general mechanisms, see sections “Modularity and Domain Specificity” and “Innateness and Development”). A fourth assumption shared by most usage-based approaches is that language can be described as a complex adaptive system, i.e., a system whose global properties emerge from multiple independent interactions of agents at a more local level (see e.g., Beckner et al., 2009).

Usage-based approaches usually focus on the cognitive organization of language in present-day speakers or on developments in the traceable history of human languages. However, it has been argued that the view of language as a complex adaptive system and the processes of cultural evolution that can be observed in language history allow for drawing conclusions about the emergence of language. For instance, Heine and Kuteva (2002, 2007, 2012) and Bybee (2010) argue that grammaticalization processes can account for the development from early (proto-)language to modern languages (see also Arbib, 2012, 2015). It has also been noted that the complex adaptive system view of language is highly compatible with those strands of language evolution research that focus on the (cumulative) cultural evolution of language (see e.g., Pleyer and Winters, 2014; Pleyer, 2017), such as the Iterated Learning paradigm that has become one of the most influential approaches in language evolution research (Kirby and Hurford, 2002; Kirby et al., 2008; Kirby, 2017).

Some usage-based linguists have put forward fairly strong hypotheses regarding the origins of language. Perhaps most prominently, Michael Tomasello, coming from a background of usage-based Construction Grammar, has proposed an elaborate theory of the evolution of language – as well as cultural cognition and species-specific symbolic behavior more generally – in the context of his shared intentionality framework (see e.g., Tomasello et al., 2005; Tomasello, 2008, 2019). Another usage-based linguist who has put forward a less broadly received (and far more sketchy) theory of language evolution is Talmy (2007), who sees the mechanism of “recombinance” as crucial for the evolution of language. By recombinance, he means “the assembly of discrete units into a new higher-level unit with its own identity” (Talmy, 2007, p. 26). As a final example, consider Keller’s (1995) monograph on historical language change, which contains a chapter proposing a Gricean theory of the evolution of the predispositions for language (but see Moore, 2017 for an updated view on the evolutionary foundations of the Gricean communicative infrastructure). These examples show that linguists coming from a usage-based framework have made fairly explicit proposals regarding the question of language origins. Researchers adopting the framework of construction grammar in particular have argued for the fruitfulness of a constructionist approach to the evolution of language (Steels, 2004; Arbib, 2012, 2015; Hurford, 2012; Johansson, 2016; Pleyer, 2017). Also, construction grammarians have adopted the generalized theory of evolution (e.g., Hull, 1988) to account for the cultural evolution of language over historical time, a particularly well-known example being Croft (2000).

### Biolinguistics

In this section, we briefly outline what we mean by biolinguistics when comparing usage-based approaches with biolinguistics. Some conceptual clarification is necessary, as the degree of consensus and dissensus of course differs depending on which sets of theories and approaches within this broad paradigm are being compared. Biolinguistics can be described as the investigation of knowledge of language within the tradition of the generative enterprise with a commitment to take into account the biological foundations of language and view it from an interdisciplinary perspective. Boeckx and Grohmann (2007) distinguish between a strong sense and a weak sense of biolinguistics. The weak sense captures the type of work that generativists have engaged in following the tradition that started with Chomsky (1957, 1965). In its strong sense, biolinguistics refers to work that explicitly integrates insights from evolutionary biology, psychology, and related disciplines. This approach can be seen as following in the tradition of Lenneberg (1967). As Boeckx and Martins (2016) point out, much of biolinguistics “has in practice been seen as a sub-field or rebranding of generative linguistics, and as such most of the work said to be biolinguistic came from there.” As such, the biolinguistic enterprise is closely related to the Minimalist Program (Chomsky, 1995) and its core tenets.

However, there is also a broader definition of biolinguistics, which Boeckx and Benítez-Burraco (2014a) have termed “biolinguistics 2.0.” Biolinguistics 2.0 can be seen as a research program whose aim is to uncover the biological foundations of language. On this view, the biolinguistics research program is not tied to a minimalist and generative view of language but characterizes a methodological approach of productively and explicitly combining research from different fields. As Di Sciullo and Boeckx (2011) state, from this perspective researchers with very different theoretical persuasions, such as Tomasello (e.g., Tomasello et al., 2005; Tomasello, 2008), can be described as doing biolinguistics (cf. Ferretti et al., 2018). This means that usage-based and emergentist researchers investigating factors such as the biological properties of the language-ready brain (e.g., Arbib, 2012, 2015), the neurological foundations of entrenchment (Schmid, 2015; Blumenthal-Dramé, 2016), the neurological foundations of semantic simulation (Bergen, 2012), or the neurological foundations of constructions (Pulvermüller et al., 2013; Goldberg, 2019), are doing biolinguistics as well. In this paper, however, our interest in convergences and divergences is somewhat more specific. Here, we want to outline similarities and differences between usage-based approaches and work that explicitly labels itself as biolinguistic, with much of it adopting a minimalist framework. In other words, we are interested in the relationship between usage-based linguistics and what Boeckx (2015, p. 436) has called the “generative/biolinguistic enterprise.”

They key commitment of the minimalist framework is the reduction of the computations and theoretical operations needed to explain language. As already mentioned in the introduction, this theoretical reduction is very much motivated by evolutionary concerns (Johnson, 2017). That is, minimizing what needed to evolve in order to make language possible can be seen as a direct reaction to the challenge of explaining the evolution of language (Johnson, 2017). In minimalism, this has been done by identifying a key conceptual component, Merge, as being central to language and its evolution (e.g., Radford, 2004; Berwick and Chomsky, 2016; Fitch, 2017). This solitary focus on Merge as the key explanandum of the complexity of the language faculty has also been criticized (Progovac, 2019). Indeed, as we are going to outline in the following sections, there have been developments in biolinguistics toward an agenda that takes other factors and domains equally seriously (e.g., Benítez-Burraco and Boeckx, 2014).

## Convergence and Divergence

In the following, we will outline some of the key areas where usage-based and biolinguistic approaches have traditionally diverged and which have been and continue to be discussed quite controversially. We will outline where we see potential convergences and where we still judge that there are (probably) irreconcilable differences between the two frameworks.

### Modularity and Domain Specificity

As Balari and Lorenzo (2016, p. 4) point out, “[t]he task of disentangling the evolutionary origins of language suffers from the lack of a consensual view about what the evolved linguistic phenotype is supposed to be.” They argue that the theoretical positions differ along two coordinates: on the one hand, language is seen as “an external, socially shared code” – on the other hand, it is viewed as “a self-contained component of the human brain.” Thus, the issues of modularity and domain specificity are partly connected with the question of “what evolved,” as, e.g., Christiansen and Kirby (2003, p. 4) and Hurford (2012, p. 173) have framed one of the most crucial questions of language evolution research. However, the key disagreements are not necessarily about what belongs to the linguistic phenotype *per se* but rather about what components of language, if any, are specific to this particular cognitive “module.” Fitch (2017) summarizes the broadly shared view that language builds upon a broad array of mechanisms shared with other species, such as concepts and categories – which underlie semantics –, voluntary control over vocalization – which underlies phonology – or sequencing and working memory, which can be seen as underlying syntax. In addition to that, he characterizes complex Theory of Mind, supra-regular grammar (i.e., a grammatical capacity that goes beyond that of so-called finite state automata, which cannot deal with more than one level of nesting; see Fitch, 2018), and complex vocal learning as “unusual human capacities.” However, there is disagreement about the extent to which such foundations of language belong to the linguistic phenotype in the strictest sense: As will become clear below, some biolinguistic approaches reduce the phenotype to the “Faculty of Language in the Narrow Sense” (FLN) as proposed by Hauser et al. (2002), while usage-based approaches tend to take a much broader view.

Bates (1994, p. 136) stresses that the logically separable issues of modularity, brain localization, and innateness are often conflated, and traditionally most approaches that see language as a module of the mind have also tended to assume an innate Universal Grammar (see section “Innateness and Development”). Still, it is important to tease these different aspects apart.

Modularity refers to the idea that the mind (partly) divides into highly specialized modules (Prinz, 2006, p. 22). Sperber (1994, p. 40) defines a cognitive module as “a genetically specified computational device in the mind/brain [...] that works pretty much on its own on inputs pertaining to some specific cognitive domain and provided by other parts of the nervous systems.” Anderson and Lightfoot (2002), who take Chomsky’s (1988, p. 133) view of language as an “organ of the mind/brain,” quite literally argue that language can indeed be seen as “a biological entity, a finite mental organ” (Anderson and Lightfoot, 1999, p. 703) and hold that UG, which they call the “linguistic phenotype,” is modular. The modules they propose include the mental lexicon and a module containing abstract compositional structures. They argue that many of the modules relevant for language are specific to language but concede that they “may or may not be separately represented in neural tissue” (Anderson and Lightfoot, 2002, p. 23).

Thus, we can distinguish two different aspects of modularity that play a role in biolinguistics: on the one hand, the idea that language is a distinct module of the mind; on the other hand, the idea that this module is characterized by modular structure in itself. Hornstein (2009, p. 5f.), for instance, accepts the former hypothesis but eschews the idea of internal modularity, arguing that a highly modular faculty of language could only have evolved *via* natural selection, which would take much longer than the 50,000–100,000 years since language first emerged according to the estimates he cites (but see Tallerman and Gibson, 2012; Dediu and Levinson, 2013, for different estimates from 150,000 to 500,000 years). Hornstein (2009, p. 8) argues that the short timespan only allows for a very small number of operations to be adapted, while the basic operations and principles of the language faculty are recruited from those that were available before the emergence of language.

This leads us to the notion of domain specificity. According to Robbins (2017), “[a] system is domain specific to the extent that it has a restricted subject matter, that is, the class of objects and properties that it processes information about is circumscribed in a relatively narrow way.” Despite the fact that modularity and domain specificity are, as per Bates’ statement cited above, logically separable entities (and as the Anderson and Lightfoot quote above shows, they are actually teased apart at times), the notion of domain specificity is often taken to refer to whether or not there is a neuronal network in the brain specialized for language (cf. Prinz, 2006, p. 24). It has to be acknowledged, though, that the concepts of module and modularity mean very different things in different contexts and disciplines (see Bates, 1994, p. 137).

This shows that the notions of modularity and domain specificity can be understood in quite different ways, which pertain to different aspects of the language-cognition interface: On the one hand, they can be considered hypotheses about the organization of language in the *brain*, in which case they are statements about the neuronal underpinnings of language. On the other hand, they can be understood as more heuristic terms describing specific functions that pertain to a specific domain (such as language) but that may still be distributed across various cortical regions.

Bates (1994, p. 139f.) even distinguishes five levels at which claims of domain specificity may apply (the task or problem to be solved; the behaviors or skills that develop to solve the problem; the representations or knowledge that must be present to solve the problem; the neural processing mechanisms required to sustain those representations; and the genetic substance that makes the aforementioned aspects possible). She argues that language can be considered domain specific at the first three levels, as it represents “a special response to a special problem” that “must be supported by a detailed and unique set of mental/neural representations.” The controversial questions, according to Bates, pertain to the question of whether we have evolved a “special form of computation that deals with language, and language alone” and if that new mechanism is biologically encoded.

This is also where opinions tend to differ between biolinguistic and usage-based approaches. The radically usage-based complex adaptive system view of language holds that language is not shaped by any domain-specific factors but rather by “[p]rocesses of human interaction along with domain-general cognitive processes” (see section “Cultural and Biological Evolution”). However, Christiansen and Chater (2008, p. 508) take a more nuanced perspective by conceding that language-specific cognitive adaptations may have occurred *via* so-called Baldwin effects, i.e., the internalization of within-generation developmental accommodation leading to evolutionary change (Badyaev, 2009, p. 1126). According to de Ruiter and Levinson (2008), it seems plausible to assume cognitive adaptations not for language but for *communication* more generally, which also raises the question of whether domain specificity may be a matter of degree both regarding the breadth of the domain to which it applies and regarding the extent of specificity. Ambridge and Lieven (2011, p. 361, 368), reviewing a wealth of studies on language development in ontogeny (including atypical development) and on the genetic basis of language, take such a gradual approach when they conclude that language can neither be completely domain general nor an entirely modular system. On the biolinguistic side, Boeckx (2012, p. 30) argues that linguistic minimalism helps overcome previous tendencies toward over-modularization, and he compares this development to a similar shift in emphasis in comparative psychology, where earlier work tended to focus on the seemingly unbridgeable gap between human language and other communication systems, whereas more recent work tends to take a bottom-up perspective that “focuses on the constituent capacities underlying larger cognitive phenomena” (de Waal and Ferrari, 2010, p. 201). In fact, Benítez-Burraco and Boeckx (2014) criticize strictly modular approaches such as that of Anderson and Lightfoot (2002) as “simplistic.” Modular approaches in general have also become more complex, so that there is more overlap with non-modular views of cognition (Barrett and Kurzban, 2006).

Overall, this more recent biolinguistic view on modularity is therefore much more in line with usage-based approaches. It is also consistent with, and informed by, neuroscientific evidence that linguistic processing might recruit other neural circuits for sequence processing, forming associations, working memory, and others (e.g., Prat, 2013; Christiansen and Chater, 2016; Gong et al., 2018; Hernandez et al., 2019).

What many biolinguists take away from such neuroscientific evidence is that language should not be seen in modular isolation but “as part and parcel of a broader cognitive basis” (Boeckx, 2017). Of course, the question to what degree language represents a neurologically domain-specific system is still intensely debated in the neuroscientific literature (e.g., Fedorenko et al., 2011; Fedorenko and Thompson-Schill, 2014; Vogel et al., 2014; Friederici and Singer, 2015; Campbell and Tyler, 2018; Dick and Krishnan, 2019). Although this debate is far from settled, the above discussion shows that different approaches are potentially converging on a more complex view of the issue of modularity. Generally, we share the view held by Boeckx and Martins (2016) that, ultimately, “modular conceptions of cognitive domains like language are likely to dissolve as we learn more about the (generic) mechanisms implementing cognition at the molecular and cellular levels.”

An important argument in favor of domain specificity in generative linguistics have been structural dissimilarities between the operations assumed to be at work in UG and what has been described for other cognitive domains (see e.g., Bates et al., 1991, p. 30). But as will become clear in the subsequent sections, the number of operations that are assumed to be part of the language faculty has been reduced substantially in current biolinguistic approaches compared to the early days of generative grammar. Hauser et al. (2002) famously distinguished a “Faculty of Language in the Broad Sense” (FLB) from the “Faculty of Language in the Narrow Sense” (FLN), the latter containing the core grammatical computations underlying language. They argued that FLN is limited to recursion, which they hypothesized to be a uniquely human and domain-specific adaptation. A complex debate (see e.g., Fitch et al., 2005; Jackendoff and Pinker, 2005; Pinker and Jackendoff, 2005; Boeckx, 2009; Watumull et al., 2014; Behme and Evans, 2015; Adger, 2015a,b, among many others) revolves around the questions of what exactly the concepts of FLN and recursion encompass and how they relate to Merge, “a process that takes any two syntactic objects (words, phrases, clauses, etc.) and joins them to form a new syntactic object” (Bickerton, 2013, p. 29). According to Berwick and Chomsky (2011, p. 30), “[o]ptimally, recursion can be reduced to Merge.” The nature of Merge is also subject to debate within biolinguistic approaches: While, e.g., Watumull et al. (2014) see it as irreducibly elementary, Boeckx (2009, p. 47) argues that it can be decomposed into more basic operations. Also, Hornstein (2009, p. 109) sees Merge as a combination of (pre-linguistic) concatenation with labeling, “an operation whereby one of the two inputs to concatenation ‘names’ the resulting concatenate” (Hornstein, 2009, p. 58). In particular, he argues that endocentric labeling, which marks one of the constituents as head, can be considered the key evolutionary innovation giving rise to unbounded recursive hierarchy.

Usage-based approaches also acknowledge the key role of recursion in human language. However, in contrast to the view of recursion as domain specific for language, emergentist approaches have suggested that recursion arises from combined activities of memory, lexicon, discourse, and role activation (MacWhinney, 2009). Christiansen and Chater (2015) propose a usage-based account of recursion, according to which the ability to process recursive structure emerges on top of domain-general learning abilities. On this view, FLN is in fact “empty,” which also calls into question the usefulness of the distinction in the first place. In fact, the FLN/FLB distinction has also been criticized within biolinguistics for directing attention away from a mosaic or composite view of language as a whole (Boeckx, 2013).

We can thus conclude that biolinguistic and usage-based approaches agree that there is a “species-specific linguistic capacity” (Benítez-Burraco and Boeckx, 2014, p. 122) and that this capacity has biological foundations.^1^ The point of contention is what exactly these biological foundations entail and to what degree they are specific to language. This can be illustrated with one of the core concepts in biolinguistics and the Minimalist Program, namely Merge. Regardless of its formal description, it is clear that some kind of process in this direction is important for language and human cognition more generally. This is also recognized in usage-based approaches: For instance, MacWhinney (2015), writing about the mechanisms of language emergence, stresses the importance of a cognitive mechanism of “composition” and explicitly remarks that “the emphasis in UG Minimalism on the Merge process (Chomsky, 2007) is compatible with emergentist accounts.” However, MacWhinney (2015) also stresses that compositionality is not a feature specific to language but is also required for non-linguistic tasks such as “basic action processing” (see also Steedman, 2004; MacWhinney, 2009; Arbib, 2015). This, then, points toward a possible divergence between usage-based approaches and biolinguistics. However, as Merge is often seen as a mechanism for combining *concepts* as well, we do see broad compatibility between usage-based approaches and biolinguistics if biolinguists acknowledge a Merge-like mechanism to operate in non-linguistic tasks such as action processing or concept formation and human hierarchical processing as well. In fact, Chomsky actually acknowledges this possibility: “Merge is one such operation that can be seen as a UG principle but also as one possibly ‘appropriated from other systems’ (Chomsky, 2007, p. 7) and relevant to other systems” (van Gelderen, 2009, p. 227).

In addition, the foundations of language are not only biological. Specifying the aspects that make the human brain “language-ready” (Arbib, 2012) is one important aspect not only of biolinguistics but also of evolutionary approaches to language more generally. Other important questions regard the evolution of the “language-ready social settings” (Pleyer and Lindner, 2014), that is, questions regarding the interactional, ontogenetic, and cultural processes that give rise to language and linguistic structure. As Balari and Lorenzo (2016) outline, much earlier work on language in the generative tradition conceived of language as a “self-contained component of the human brain” (4) that is modular and domain specific. However, they stress that there is currently a shift toward “a composite or mosaic conception of language” (see also Boeckx, 2017). Strong domain specificity is therefore demoted in recent biolinguistic theorizing. This means that in this domain, biolinguistics has moved closer to the position held in usage-based approaches.^2^ In fact, the metaphor of a “mosaic” (Wang, 1982) view of the language-ready brain can be found in both biolinguistics (Benítez-Burraco and Boeckx, 2014; Boeckx, 2017) and usage-based and emergentist, domain-general approaches (Gong et al., 2018) to language and its evolution.

### Innateness and Development

In this section, we want to focus on two key areas where we see a potential for convergence between biolinguistics and usage-based approaches. The first concerns changing conceptions of UG and the role of innate, domain-specific biological foundations of language within biolinguistics. The second concerns the growing importance in both biolinguistics and usage-based theories of more refined and complex views of the dynamic, interactive relationship of biology, development, environment, and evolution (e.g., Benítez-Burraco and Longa, 2010; Benítez-Burraco and Boeckx, 2014; MacWhinney, 2015). We will discuss each of these issues in turn.

In the traditional generative view, notions of innateness, Universal Grammar, and the poverty of stimulus argument are of central importance. According to the traditional view, following Chomsky (e.g., Chomsky, 1988), external language data are not sufficient for children to constrain the hypothesis space of how language works. The proposed solution for this problem was linguistic nativism: the child has to come equipped with prior innate knowledge of certain features of language in order to be able to learn language. These language-specific biological foundations of the language faculty have become known under the term Universal Grammar, or simply UG. Much of the work in generative grammar was done with the aim of specifying what is part of UG. We support Boeckx and Benítez-Burraco’s (2014b) use of “language-ready brain” as an alternative to UG and “language organ,” as we agree that these terms “have come to be seen as too ideologically loaded.” However, we are not sure if UG as such an entrenched concept – albeit one that is in constant change and in permanent definitional turmoil – will vanish from the biolinguistic literature anytime soon. For this reason, we think that it is worthwhile to explicate how the perspective of UG minimalism, or a biolinguistic UG, differs from usage-based approaches in regard to some core issues.

The assumption of a domain-specific innate Universal Grammar, “language organ,” or “language acquisition device” has traditionally been the single most important difference between the approaches outlined above. Discussions have long revolved around the question of “what is innate and why” (Putnam, 1980). What these approaches have in common is that they agree that language acquisition builds on biological foundations. The question, however, is to what extent these capabilities are language-specific. As shown in section “Modularity and Domain Specificity,” these terms are construed in different ways not only across different theoretical frameworks but also within minimalist/biolinguistic accounts. Meanwhile, the concept of UG continues to be hotly contested.

Proponents of usage-based approaches tend to evoke Occam’s razor (e.g., Everett, 2016): If there is no need to assume an innate UG, we should drop the assumption in order to arrive at a leaner theory. As Tomasello (2003, p. 304) puts it, “[w]hy do we need the phlogiston/ether of universal grammar (...) at all? What is it doing anyway? Why not just chuck it?” From a usage-based perspective, domain-general mechanisms can fully account for virtually all aspects of language emergence, acquisition, and use, which is why UG is seen as theoretical ballast that should be shed, unless there are compelling arguments in its favor. Bickerton (2013, p. 110), by contrast, argues that a dedicated adaptation for language “should be the null hypothesis, and the burden of proof should lie on those who challenge it” (see also Wunderlich, 2004).

Much of the work taking a UG perspective on language was done in the Principles and Parameters framework (e.g., Lohndal and Uriagereka, 2014). In this framework, UG was taken to consist of principles covering structural features shared by all languages, as well as parameters, whose settings were fixed by external data from individual languages, akin to a switch being flipped into one position or another. However, with the advent of the minimalist program (Chomsky, 1995), the P&P framework has become less and less popular as the new goal of generative research, as outlined above, was reducing UG to its minimum requirements. This has led many biolinguists to reject the P&P framework (Boeckx, 2015, p. 435; see also Dąbrowska, 2015).

The minimalist conception of language acquisition has led to a number of tensions within generative approaches to language acquisition as well as to a number of problems, as discussed in detail by Longa and Lorenzo (2008). For one, it is unclear what the status of the poverty of stimulus argument is in biolinguistics and minimalism (Longa and Lorenzo, 2008). In usage-based approaches, as well as in others, the poverty of stimulus argument has come under quite intense criticism (see e.g., Pullum and Scholz, 2002; Tomasello, 2004; Clark and Lappin, 2011; Dąbrowska, 2015). Usage-based and emergentist approaches have instead concentrated on the question of how language arises from usage through processes of generalization and self-organization (e.g., O’Grady, 2008; MacWhinney et al., 2014). These approaches emphasize that the input learners receive is actually quite rich and that distributional and item-based learning strategies are highly effective ways of learning complex linguistic structures (cf. Tomasello, 2003; Clark, 2015; MacWhinney, 2015). This remains a hotly debated topic. For example, Perfors et al. (2006) proposed a Bayesian model of grammar induction able to learn syntactic structures without the need for language-specific biases. This and other models, in turn, were criticized by Berwick et al. (2011), who reiterated the need for innate, domain-specific factors in accounting for language acquisition. However, in line with our reasoning in this paper, Berwick et al. (2011) state that in principle they share similar goals with usage-based, emergentist, and general cognitive approaches: “we share the desire to reduce any language-specific innate endowment, ideally to a logical minimum” (Berwick et al., 2011, p. 1210).

Overall, conceptualizations of UG continue to evolve, and more recent formulations of what UG is can be argued to be more consistent with usage-based and emergentist theorizing (cf. Mendívil-Giró, 2018). Many researchers in biolinguistics acknowledge that UG could in fact not contain domain-specific and language-specific properties. For example, Roberts and Holmberg (2011) acknowledge that “UG does not have to be seen as either language-specific or human-specific” (quoted in Dąbrowska, 2015). This possibility is also explicitly acknowledged by Fitch et al. (2005) when talking about the distinction between FLB and FLN: “The contents of FLN are to be empirically determined, and could possibly be empty, if empirical findings showed that none of the mechanisms involved are uniquely human or unique to language, and that only the way they are integrated is specific to human language.” This is consistent with the desire shared by minimalists, biolinguists, as well as usage-based and emergentist approaches to reduce what is specific to language as much as possible. In fact, the “minimalist” desire to try to attribute as little as possible to language-specific biological prerequisites is shared by many other approaches as well (Haspelmath, 2017).

One other key point of potential convergence between usage-based approaches and biolinguistics is the increasing acknowledgment that ontogenetic development should be seen in terms of a complex adaptive system in which multiple factors interact. This has direct consequences for conceptions of innateness, nativism, and the nature-nurture debate that have plagued the field for such a long time.

If complex traits like language emerge from the dynamic interactions of different factors at different timescales, this also means that “[a]sking whether a particular principle is “innate” or due to “external stimuli” is meaningless – it is both” (Dąbrowska, 2015, see also Mendívil-Giró, 2018). This is also echoed in recent biolinguistic publications. For example, Bowling (2017) stresses that separating cultural and biological contributions perpetuates a false dichotomy between nature and nurture. In biolinguistics, this focus on the developmental dynamics of language is often seen in the context of evo-devo, with the proposal that concepts from (ecological) evolutionary developmental biology such as developmental plasticity, robustness, and canalization (e.g., Gilbert and Epel, 2009; Pigliucci and Müller, 2010; Laland et al., 2015) might play an important role in explaining language emergence as well as its variation and variable acquisition (e.g., Benítez-Burraco and Boeckx, 2014, p. 126). From an evo-devo perspective, it

is not possible to distinguish relevantly between the influence of the genes and the influence of the environment in development, since the end product is the result of the interaction of the information from both levels. In light of Evo-Devo, few dichotomies (e.g. I-Language/E-Language, Nature/Nurture, FLN/FLB, gradualism/saltationism and even adaptation/exaptation) make perfect sense (Martins et al., 2016, p. 161).

Evo-devo also goes along with a reconceptualization of the concept of gene. In an interactive perspective, it has become clear that “[g]enes are not blueprints” (Benítez-Burraco and Boeckx, 2014, p. 125). They “do not encode specific behaviors, cognitive processes, or even neural circuits, they make proteins that interact in complex, environmentally modulated networks, to build and maintain brains” (Bowling, 2017; cf. Fisher, 2006, 2017). Thus, biolinguistics is moving past simple genetic determinism, leading to common ground with usage-based approaches. However, as biolinguistics is not a unified field but more of a program or enterprise, as outlined in section “Biolinguistics,” this view seems not to be the consensus in biolinguistics yet. In fact, much of the literature is still dominated “by naive depictions of the biology of language” (Benítez-Burraco and Boeckx, 2014). For example, Benítez-Burraco and Longa (2010) take Chomsky (2010) to task for advocating a simplistic and deterministic genetic view of evo-devo that does not do justice to the complexity of more dynamic evo-devo approaches (Benítez-Burraco and Boeckx, 2014, p. 124). In the context of language evolution, Bowling (2017) also criticizes the view according to which language emergence must be explained with reference to specific genetic modifications – a view espoused, for example, by Bolhuis et al. (2014). It has to be noted, though, that Bowling (2017) also criticizes, e.g., Kirby’s (2017) and colleagues’ work on iterated learning (see section “Cultural and Biological Evolution” below) for not taking developmental processes and gene-culture interactions seriously enough. Thus, it is fair to say that at least some of the work in usage-based and emergentist approaches still needs to properly integrate research from evo-devo and developmental systems theory. Overall though, as Dąbrowska (2015) points out, “it is encouraging to see the two traditions in cognitive science are converging, to some extent at least.”

However, this is not to say that questions of innateness are not still prevalent in debates between generativists and usage-based theorists. For example, Adger (2013, 2018) claims that usage-based approaches are, in the words of Quine (1969), “knowingly and cheerfully up to [their] neck[s] in innate mechanisms of learning readiness.” He claims that usage-based and cognitive-linguistic theories of language use such as Talmy (1975, 2000), Langacker (1987), and Goldberg (2006) presuppose innate mechanisms and therefore simply reject one form of innateness, namely language-specific innateness, for another one, domain-general innateness. Such criticisms do not take into account the need for a more complex and dynamic perspective on ontogenetic processes. This point is also made by Goldberg (2013) in her response to Adger’s (2013) criticism: “Constructionists generally do not make any claims about whether these other biases are ‘innate’ since the term woefully underestimates the typically complex interactions between genes and the environment before and after birth.”

Indeed, if an evo-devo and complex adaptive systems approach to development is taken seriously, it might be time to discard the concepts of innateness and maturation altogether, a position taken, for example, by Overton (2015). As he writes, “any characteristic is the outcome of a long and continuous epigenesis entailing embodied activities and actions (experiences), beginning at conception and continuing through prenatal and postnatal phases of development, as well as across the life span” (see also Bateson, 2015; Lickliter and Honeycutt, 2015). This renders concepts such as innateness meaningless and possibly even counterproductive as they do not take into account the importance of experience and environmental factors. To move past the concept of innateness, both biolinguistics and usage-based approaches need to properly acknowledge that development is always scaffolded in myriad ways (Caporael et al., 2014; see also Balari and Lorenzo, 2016) and that there are complex interactions within developmental systems. Development takes place in a particular evolutionary niche shaped and geared toward scaffolding learning processes. This ranges from the structure of interactions available to learners to symbolic artifacts that scaffold learning. These environments in turn are also shaped by learners themselves. Moreover, the emergence of particular learning factors in turn scaffold subsequent development in cascading, dynamic feedback loops within a multidimensional developmental web (e.g., Karmiloff-Smith, 2009; Caporael et al., 2014; Mascolo and Fischer, 2015; Overton, 2015; Carpendale et al., 2018).

Two domains where the growing importance of evo-devo and complex system considerations have direct impact on linguistic theorizing are the issue of modularity, discussed in section “Modularity and Domain Specificity” above, and the question of a critical period of language learning.

In traditional generativist accounts, a critical period was directly linked to concepts of an innate language faculty and its genetically determined maturation. On this view, which is still held by many researchers in a generative framework (see e.g., Lust, 2006, p. 93), there is a “time-window,” a critical period, in which experience can trigger the development of the language faculty. Outside of this critical period, language acquisition might be severely impaired or hindered. Many usage-based and emergentist language acquisition researchers have long preferred to talk about sensitive instead of critical periods for language learning (e.g., Rowland, 2014). A decline in language learning abilities is framed in terms of an interplay of social factors – such as different types of and less rich interactions –, cognitive factors – such as entrenchment and competition with first language structures –, as well as biological factors such as reduced neuroplasticity (e.g., MacWhinney, 2012).

However, recent theorizing in biolinguistics has significantly reformulated the critical period concept in a way that makes it much more compatible with usage-based and emergentist approaches (Balari and Lorenzo, 2015). Balari and Lorenzo (2015), for example, argue that the way that critical periods are being talked about in generative approaches often does not take into account that language is a complex developmental phenomenon. They argue for a conception of language not as a faculty but as a “gradient,” i.e., an aggregate of cognitive abilities, the weight of which is variable from one to another developmental stage, and which exercise crucial scaffolding effects on each other (Balari and Lorenzo, 2015). Of course, both approaches agree that there are age effects in language acquisition (see e.g., Werker and Hensch, 2015; Blom and Paradis, 2016). However, in contrast to the traditional maturation-trigger model, more recent approaches have taken a much more dynamic, interactive, complex systems view of this complex relationship, which can be seen as offering potential for finding common ground between the approaches. Of course, many researchers continue to talk of critical periods, critical period effects, and maturation (e.g., Werker and Hensch, 2015). However, these approaches still share a dynamic conception of age of acquisition effects with usage-based and emergentist approaches. Werker and Hensch (2015), stress that conceptions of critical periods are in a constant process of being modified to take into account the dynamic interplay of experiential and maturational influences that lead toward a trend for system stability and the fact that critical period effects themselves exhibit features of plasticity. That is, work on age of acquisition effects from different traditions can help specify the processes that mediate, narrow, and reopen learning processes (cf. Bavelier et al., 2010).

The evo-devo and complex systems perspective also has implications for conceptualizations of modularity, which were discussed in detail in section “Modularity and Domain Specificity.” From this perspective, possible domain-specific effects can be captured by the concept of developmental modularity (Karmiloff-Smith, 1992). This view is encapsulated in Bates et al.’s (1988, p. 284) dictum that “[m]odules are not born, they are made.” For example, Hernandez et al. (2019) propose a neuroemergentist framework in which complex functions such as language arise out of the interactional dynamics of pre-existing neural mechanisms which have evolved for different functions. These then become recycled and restructured and self-organize into new networks, yielding apparent functional specialization. This view is also consistent with research showing that weak, domain-general biases can have domain-specific effects (Culbertson and Kirby, 2016).

In evolutionary terms, these considerations are in line with the position that evolution is a “tinkerer” combining existing systems to yield new functions (Jacob, 1977; Gong et al., 2018). They are also consistent with the view that, as Bates et al. (1991, p. 34) put it, “[l]anguage is a new machine built out of old parts,” with the old parts, however, keeping “their day jobs” (Bates, 1999, p. 237). This perspective also takes seriously the fact that many different developmental trajectories can lead to the emergence of language and that language can be quite variable developmentally, cognitively, as well as neurobiologically (see also Benítez-Burraco and Boeckx, 2014, p. 124). Developmental modularity therefore sees modularity as being an emergent, permeable, and interactive process leading to robust and reliable development *via* variable pathways and through variable system implementations (McClelland et al., 2006; MacWhinney, 2015). In fact, if developmental modularity is framed in this way, the discussion can move away from all-or-nothing choices regarding modularity and toward the factors that influence the emergence of relatively stable and specialized functional neurobiological systems (cf. Barrett and Kurzban, 2006).

One interesting question is to what extent the emergence of co-opted, recycled functional systems had co-evolutionary, emergent effects. This is also explicitly acknowledged by usage-based linguists such as Dąbrowska (2015), who states that the “old parts” such as attention, motor planning, and memory consolidation evoked by Bates (e.g., Bates, 1999) might have “undergone further selection as a result of the role they play in language, so that language is now their ‘day job,’ although they continue to ‘moonlight’ doing other jobs.” This is also echoed in the biolinguistic literature. Boeckx (2017, p. 327), for example, states that

[o]f course, once collected under a single roof (“language-ready brain”), these traits may give rise to nonlinear, “emergent” effects. Likewise, as Fujita (2016) has stressed, when placed in the context of the human brain, “old” pieces may acquire new roles that transform their nature (the sort of feedback loop familiar in biology).

Their linguistic recruitment might therefore in turn influence the biological evolution of domain-general constraints such as brain size, memory load, storage capacity, and patterns of neural development, perspective-taking, and sociocognitive skills, among others. This co-evolutionary relationship between language and the biological foundations adapted by language is explicitly acknowledged in both usage-based approaches and biolinguistics (Gong, 2011; Hurford, 2012; Steels, 2012).

These considerations move away from the ontogenetic process and more toward an integrated evolutionary account of ontogeny, culture, and biology, which is the topic we are going to turn toward next.

Overall though, although we see a potential for increasing dialogue and convergences between usage-based approaches and biolinguistics, we agree that the question of how the language system “specializes and the extent to which it interfaces with evolutionarily conserved processes needs to be much better understood mechanistically and across neural scales” (Petkov and Marslen-Wilson, 2018). In addition, as Fitch (2017) points out, many of the issues discussed in this section are “not likely to be resolved until we know more about how genes, brains, and language are interrelated.”

### Cultural and Biological Evolution

The complex adaptive system view on ontogenetic development described in the previous section can be related to a broader complex adaptive system view of the relationship between ontogeny, cultural, and biological evolution (e.g., Beckner et al., 2009; Steels, 2011; Pleyer and Winters, 2014; MacWhinney, 2015; Kirby, 2017). After focusing on ontogeny in the previous section, in this section, we will spell out possible convergences and differences between biolinguistics and usage-based approaches in the domains of biological and cultural evolution. Here the complex adaptive systems view as a framework opens up new possibilities of dialogue about the factors that influence the emergence of language across multiple timescales. As Bentz (2018) points out, the complex adaptive system approach functions as an overarching framework and can accommodate both strong minimalism and usage-based theories of language.

However, apart from the adoption of an overarching framework that enables more fruitful dialogue, there are also other developments that bring biolinguistics and usage-based theory closer together. As outlined in the previous section, biolinguists and minimalists have realized that they made too heavy demands on the genetic endowment required for language (Boeckx, 2017; see also O’Grady, 2012). We have already seen in the previous sections that much of the developmental “burden” of UG has been shifted to other factors. This holds not only for the ontogenetic level but also for the development of language across multiple timescales, as well. Chomsky (2005), for example, has proposed that next to genetic endowment as a first factor, and experience as a second factor, there is a third factor contributing to language design, namely “principles not specific to the faculty of language.” Some authors, such as O’Grady (2012), cautiously treat this concept as offering the potential for convergence with usage-based approaches, while others remain extremely skeptical (e.g., Johansson, 2013). Usage-based and emergentist approaches have concentrated on the question of how language arises from multiple competing constraints, such as usage and processes of generalization and self-organization (e.g., MacWhinney et al., 2014; MacWhinney and O’Grady, 2015). As O’Grady (2012) puts it, “[b]roadly speaking, the rest of the field has been committed to the primacy of third-factor explanations for decades.” As he points out, the fact that minimalism and biolinguistics show an increasing interest in “third factor principles” offers the “opportunity – the first in half a century – for the discipline to focus on a common research question: What are the nongrammatical mechanisms and forces that shape language and contribute to its effortless acquisition?”

Even though we can observe convergences between biolinguistics and approaches that stress the cultural component of the emergence of language (cf. Boeckx, 2017), the central question remains how much of language can be explained in terms of cultural evolution.^3^ Regarding the importance of the cultural dimension of language emergence, there is a wealth of research in grammaticalization research which shows that structure and complexity emerge historically through processes of language change (e.g., Heine and Kuteva, 2007). Some approaches therefore assume that the evolution of language can be explained exclusively by recourse to cultural evolution. For instance, in Steels’ recruitment theory, “genetic evolution by natural selection is not seen as the causal force that explains the origins of language” (Steels, 2007, p. 131). Instead, other, domain-general cognitive and neural resources are “recruited” for communication (Steels, 2007, p. 130). Other approaches do not rule out the existence of innate, language-specific mechanisms entirely but still emphasize the key role of cultural evolution (see Hurford, 2012). For instance, Kirby (2017), in line with the complex adaptive systems approach, posits that “[w]ith a trait like language, biological evolution takes place alongside individual learning and cultural transmission.” The iterated learning paradigm adopted by Kirby and colleagues (e.g., Kirby and Hurford, 2002; Kirby et al., 2008; Kirby, 2017) is one particular approach to the cultural emergence of structure that is highly relevant to evolutionary linguistics. In a number of computational modeling studies as well as in experimental studies, iterated learning research has shown that structured communication systems as well as linguistic structure can emerge through iterated learning. The learning biases of learners exposed to unstructured input over time lead to the emergence of structure if the second generation of learners is exposed to the output of the first generation, and the third generation is exposed to the output of the second generation of learners, and so forth. Linguistic structure can therefore be said to emerge from repeated and iterated cycles of usage and learning. Adger (2017) sees these results as consistent with generative grammar. As he states, such results are in line with Chomsky’s (2005) view of third factor effects. Adger (2017) interprets the emergence of structure through Bayesian Iterated Learning as resulting from “general laws of computational economy,” which interact with social and cultural pressures. In his view, it is still important to note that such changes still take “place within the constraints imposed by the nature of the human language capacity itself.” This is echoed by O’Grady (2012), who states that both usage-based and emergentist approaches on the one hand as well as minimalist and biolinguistic approaches on the other must look toward “yet-to-be-discovered constraints on processing, perception, cognition, and interaction” that shape human language.

Bentz (2018, p. 25) makes a similar point by stating that results from iterated learning might indeed be consistent with minimalism, as iterated learning explains the origin of structure in language, whereas minimalism is interested in the core computational features which make the computation of such structure possible in the first place. Usage-based and biolinguistic approaches seem to differ regarding the aspects of the emergence of language they focus on. The emergence of structure is of course constrained and based on the properties of the “language-ready brain,” but many usage-based theorists also emphasize the fact that the structures of languages adapt to and are shaped by the brain (e.g., Christiansen and Chater, 2008, 2016) as well as by social, communicative, and processing factors. With increasing biolinguistic forays into “third factor principles,” however, there is more potential for both approaches to enter into a dialogue with regard to the factors that shape language. From this perspective, both approaches can deal with the question of “which aspects of language in a usage-based sense are potentially to be explained by factors external to FLN, and maybe even external to FLB?” (Bentz, 2018, p. 26).

Recent variants of the iterated learning paradigm have taken the connection between culture and biology into account more thoroughly, partly in response to the frequent criticism that the individuals involved in the lab experiments are fully modern humans. Thompson et al. (2016) propose a series of Bayesian computational models of gene-culture coevolution and arrive at the conclusion that “[c]ulture facilitates rapid biological adaptation yet rules out nativism: Behavioral universals arise that are underpinned by weak biases rather than strong innate constraints.”

In general, then, the importance of cultural evolution and non-biological factors in the emergence of language is acknowledged in biolinguistics, and both approaches might find common ground in investigating these factors. In fact, Gong (2011) argues that biolinguistics can help in identifying biological constraints on language structure and in evaluating their role in language evolution. He explicitly argues that biolinguistics and evolutionary linguistics can meet in tackling the question of how biological constraints are differentially recruited in language evolution and learning.

One caveat that has to be noted here, though, is that the emphasis of much of minimalist biolinguistics lies less on general cognitive and social factors in explaining the emergence of language. Instead, minimalist biolinguistics tend to stress the importance of more abstract, computational principles. Chomsky (2005), for example, divides third factors into the following subtypes:

(a) principles of data analysis that might be used in language acquisition and other domains; (b) principles of structural architecture and developmental constraints that enter into canalization, organic form, and action over a wide range, including principles of efficient computation, which would be expected to be of particular significance for computational systems such as language.

Whereas much of usage-based theory, as seen above, has focused on the effect of specific cognitive mechanisms as well as interactional, communicative, and social factors, Chomsky (2005) stresses that it is the second subcategory that is expected to be much more significant in explaining language emergence. This raises problems for finding common ground with usage-based approaches on two levels.

First, as noted by O’Grady (2012), computational efficiency is very much a theory-internal concept. A minimalist analysis of a given linguistic phenomenon looks very different from the analysis of the same phenomenon from a construction grammar perspective or from analyses in other linguistic frameworks (see Müller, 2018 for an extended discussion). This is especially so as computational efficiency in a minimalist framework is not the same as processing cost, as minimalism still upholds the competence/performance distinction, a position that is rejected in usage-based approaches. We will outline this fact in section “Knowledge of Language and Its Description” below. Therefore, if one adopts a minimalist framework that does not enter into contact with biological and psycholinguistic considerations, it is not possible to independently and interdisciplinarily test assumptions about the influence of third factor principles without also taking on board the assumptions of minimalism (O’Grady, 2012). Of course, to a degree this presents a general challenge for all theoretical linguistic approaches that appeal to computational efficiency. This point is also made by Fitch (2017), who notes that computational simplicity “does not necessarily translate into implementational simplicity at the neural level (or vice versa)” (see also Poeppel and Embick, 2005).

Second, the minimalist focus on “more general principles that may well fall within extra-biological natural law” (Chomsky, 2011, p. 263) has been criticized for being too vague and ultimately unhelpful in capturing the factors involved in language emergence (Johansson, 2013). Johansson (2013), in his critique of the third factor concept, argues that there is no clear consensus in biolinguistics on how to approach the question of what might count as a third factor principle, making appeals to third factors a “vague and disparate collection of unrelated components.” Moreover, he criticizes the often sweeping references to physics without principled explanations. Speaking of generalized third factor principles might therefore be much less productive than proposals of specific factors of a non-linguistic nature that influence the emergence of language. It is this potential for debating specific factors influencing language design where we see the greatest potential for cross-fertilization between the approaches.

One prominent usage-based approach relevant to this discussion is that of Christiansen and Chater (2008, 2016), who argue that “language is shaped by the brain.” That is, they argue that language emergence was driven by linguistic structure adapting to the non-linguistic mechanisms and constraints that operate when generations of language users learn and process language in real time. Specifically, they point to the pressure deriving from multiple interacting constraints that shape language. These constraints belong to a number of different domains, namely

1. the nature of the cognitive activities and thoughts language is used to express,
2. constraints from the perceptual and motor machinery underlying language,
3. cognitive constraints on learning and processing such as memory constraints and constraints from processing sequential and hierarchical structures, and
4. pragmatic constraints.

Deacon (1997, 2012) takes a similar approach to constraints on language structure. He breaks down such constraints into four main categories, which partly overlap with those proposed by Christiansen and Chater (2008), but in part extend them as well: (1) semiotic constraints on the structure inherent in a referential symbolic system, (2) processing constraints that enable language processing to be automatized, (3) phylogenetic sensorimotor biases relating to the embodied nature of language and conceptualization (see also Lakoff, 1987; Langacker, 1987; Hurford, 2007; Johnson, 2018), and (4) communicative constraints relating to the way and types of information shared in human societies. Although much of minimalist biolinguistics has been more interested in what the core features of language are, it can be argued that it is crucial to focus on the question of what kinds of constraints shape the emergence of language to get a clear picture of what the core features of language are. In fact, many biolinguists agree that the deep systematic constraints on language are a central factor in accounting for the emergence of language in all its variation that is not only consistent but also very much in line with an evo-devo approach to language (e.g., Benítez-Burraco and Boeckx, 2014).

Of course, the key questions for the future will be to what degree such factors can explain the emergence of language and what picture of the structure of “the language-ready brain” emerges from these investigations. Fitch (2017), for example, agrees “with Keller (1995), Deacon (1997), Heine and Kuteva (2002), Steels (2017), Kirby (2017), and many others that much of the complexity evident in the syntax of modern languages has arisen repeatedly by grammaticalization processes of cultural evolution and required no further neural changes beyond those needed for dendrophilia,” a domain-general ability to process and perceive hierarchical structure, which evolutionarily came to be applied to language and other hierarchical behaviors such as music and art.

However, the debate about the role of grammaticalization and cultural evolution is still ongoing. This is also related to the notion of protolanguage. Whereas the notion is rejected outright by minimalist approaches that take a saltationist view, as the emergence of unlimited Merge is seen as the *sine qua non* of any form of “language” (e.g., Berwick and Chomsky, 2016), others have proposed quite detailed models of protolanguage stages, which are also rooted in evolutionary changes (e.g., Jackendoff, 2002; Progovac, 2015). Usage-based and emergentist approaches, such as that of Arbib (2012, 2015) and Heine and Kuteva (2002, 2007, 2012), on the other hand, differ from these approaches in that they assume that processes of cultural evolution and grammaticalization can lead from a protolanguage stage to language without any further biological changes. This perspective in turn is criticized by researchers in biolinguistics, many of whom accept a protolanguage stage, but, in contrast to Arbib (2012, 2015), “while recognizing the importance of cultural learning and transmission, still allow for significant changes at the level of the brain between a protolanguage user and a full-fledged, modern-language user” (Boeckx, 2017). This view, it is argued, is consistent with recent research indicating that there have been changes to the human brain even after the emergence of modern humans, which might have influenced the cognitive mechanisms involved in the process of grammaticalization (Benítez-Burraco, 2017).

So while there clearly are convergences regarding the recognition of the importance of cultural evolution between usage-based approaches and biolinguistics, the relation between cultural and biological evolution in the emergence of language is in need of further exploration.

### Knowledge of Language and Its Description

Another contested topic that is closely related to – and immediately follows from – the issues discussed above is the relative importance of competence and performance, or “I-language” and “E-language.” It has often been noted that the various terms that have been suggested for these different facets of language are not fully congruent: For instance, Jackendoff (2002, p. 29) points out that while I(nternalized)-language “coincides more or less with competence,” E(xternalized)-language does not refer to “the mechanisms that speakers use to exhibit linguistic behavior (i.e., performance), but either (a) external linguistic behavior of individuals or (b) language regarded as an object external to human minds.” He also notes that Saussure’s *langue* and *parole* both correspond to aspects of E-language.

From a minimalist perspective, the study of language amounts to the study of I-language. The term “I-language,” introduced by Chomsky (1986), in essence refers to “the linguistic knowledge in the head of a native speaker, that is, the grammar” (Culicover, 2013, p. 194). Interestingly, this definition could also be applied to what construction grammarians have termed the “construct-i-con.” According to Hilpert (2013, p. 1), summarizing Goldberg (2003), the main objective of Construction Grammar is “to find out what speakers know when they know a language and to describe this knowledge as accurately as possible.” However, the answer to this question trivially depends on whether one assumes an I-language in the first place.

Usage-based approaches do not usually distinguish between I-language and E-language. This is not to say, of course, that they do not make a distinction between language as an externalized, “materialized” phenomenon, and its cognitive underpinnings. But while generative approaches hold that the properties of I-language cannot be derived from the observable facts of E-language (see e.g., Anderson and Lightfoot, 2002, p. 9), usage-based approaches put the study of E-language center stage, arguing that linguistic usage patterns allow for important conclusions regarding the cognitive organization of language (see e.g., Bybee, 2010; Taylor, 2012).

The question of whether we have to distinguish between I-language and E-language (or make related distinctions) also entails important epistemological and methodological consequences. Adli et al. (2015, p. 10), discussing points of convergence and difference between generative syntax and variationist sociolinguistics (which tends to be conducted in a usage-based framework), phrase the main issue quite succinctly: “In essence, the question is whether grammar contains numbers or not?” In other words, the question is what, if anything, we can learn from usage data about language as a (cognitive) system. According to Taylor’s (2012) “mental corpus” hypothesis, which is heavily influenced by other usage-based approaches (especially the works of Bybee and Langacker, e.g., Langacker, 1987; Bybee, 2010), language users keep track of the utterances they encounter, which leads to the (ontogenetic) emergence and lifelong reconfiguration of a network of linguistic constructions. From this perspective, the cognitive organization of language can be fully understood by describing E-language. This is why usage-based constructionist approaches posit that language can be exhaustively described in terms of constructions, that is, form-meaning pairs at various levels of abstraction (Croft, 2001; Goldberg, 2006) or, in Goldberg’s (2019, p. 7) most recent definition, “emergent clusters of lossy memory traces that are aligned within our high- (hyper!) dimensional conceptual space on the basis of shared form, function, and contextual dimensions.” However, it has increasingly become clear to proponents of usage-based approaches that a direct mapping from usage to cognition is not possible. For example, Dąbrowska (2016, pp. 486–488) sees the corpus-to-cognition fallacy, i.e., the assumption “that we can deduce mental representations from patterns of use,” as one of the “seven deadly sins of Cognitive Linguistics.” This may be seen as an indication that usage-based approaches have become less radically usage-based in the sense that they have become more cautious regarding an apodictic identification of “grammar” (or, perhaps more generally: linguistic knowledge) with “usage.”

Still, the conceptualization of grammar (and its relation to usage) differs considerably between the two approaches. The difference between the holistic stance taken by usage-based approaches and the modularistic stance taken by minimalist ones is reflected in different scientific metaphors used to describe how language makes use of finite means to create a potentially infinite array of different utterances. Abrahamsen and Bechtel (2012, p. 14) describe the Chomskyan “rules-and-representations” approach to language that has proven influential not only in generative linguistics but also in cognitive science more generally as an instance of the so-called computer metaphor (see e.g., Boyd, 1993; Johnson and Rohrer, 2007; Hartmann, 2015). In line with the idea that cognition consists of representations and rules that combine them, generative approaches typically assume a strict distinction between the lexicon as an inventory of elements that cannot be derived on the basis of rules, on the one hand, and the grammar as a set of rules for combining these elements, on the other. Taylor (2012) calls this the “dictionary-and-grammar-book” approach.

Usage-based approaches, by contrast, have proposed a dynamical systems view of the mind (Spivey, 2007, p. 305). On this view, as outlined in section “Modularity and Domain Specificity,” we cannot strictly distinguish between different cognitive “modules,” let alone between different subsystems of language. Instead, “[e]verything is connected” (Beckner et al., 2009, p. 18). While the distinction between grammar and lexicon remains an important heuristic device in usage-based linguistics – especially in approaches to grammaticalization, many usage-based theorists assume a continuum between lexicon and grammar (termed “lexicon-syntax continuum” in constructionist approaches; see e.g., Broccias, 2012; Hoffmann and Trousdale, 2013; but see Pulvermüller et al., 2013 for some caveats from a neurolinguistic perspective). This entails a unified approach to the description of linguistic units – lexical as well as grammatical – on various levels of abstraction. As Hilpert (2013, p. 2) puts it, “[a]ll that speakers need to have, according to the constructional view, is knowledge of constructions.” In a similar vein, Langacker’s (e.g., Langacker, 2008) Cognitive Grammar limits the descriptive apparatus to semantic, phonological, and symbolic structures.

Given the holistic outlook of usage-based approaches, their conceptualization of how complex units are formed differs from the one in minimalist approaches: Usage-based and emergentist approaches often prefer the concept of “schemas” over that of rules. Interestingly, Booij (2010, p. 5), who combines Goldbergian Construction Grammar with a Jackendoffian Parallel Architecture approach in his Construction Morphology, sees the difference as merely terminological:

Jackendoff uses the term “rules” for regularities on a particular level of linguistic description, such as phonology or syntax. However, nothing hinges on this term, and one could use the term “schema” here as well.

However, one could also argue that the use of “rules” vs. “schemas” entails a fundamental difference in conceptualization. According to Michaelis (2012), “[a] leading insight of CxG from its inception is that grammar rules are not procedures but category descriptions, and as such, subject to taxonomic organization. Such taxonomies, which have come to be known in the CxG literature as inheritance networks, provide for cross-cutting generalizations about constructions.” Therefore, inheritance networks and different levels of abstraction and multicomponential type instantiations are the theoretical terminology used in CxG instead of “rules.” Similarly, Langacker (1987) analyzes grammatical “rules” as symbolic units that are both complex and schematic. So in terms of how the language system works, there is a deep divide between usage-based and emergentist and biolinguistic approaches. This also relates to computational approaches and the computational theory of mind, which is rejected in usage-based and emergentist approaches. However, ultimately, biolinguistic approaches are interested in conceptualizing language in terms of neural oscillation patterns and spiking activation in brain circuitry. In usage-based and emergentist approaches, this is also what schematizations eventually boil down to, meaning that even though there is a terminological difference between “rules” on the one hand and “networks” and “schemas” on the other, this terminological difference might actually be less important once we get to the granularity of neuronal activation patterns and neural implementation generally. Note also the so-called granularity problem, which refers to the fact that theoretical concepts in linguistics and neuroscience do not match, which at the moment might still present a challenge for both approaches (Poeppel and Embick, 2005; Shay et al., 2017).

In sum, then, the theoretical conceptions of linguistic knowledge still differ quite considerably between both approaches, which entail methodological differences in the sense that (externalized) language data are interpreted in different ways. Thus, the biology of language looks quite different if seen through a minimalist-biolinguistic lens, compared to the conceptualization of language from a usage-based perspective. In particular, it is an open question to what degree actual usage data can give clues to the underlying biology of language. Also, there are many open questions regarding the neuronal basis of language and the degree to which it is compatible with theoretical assumptions and concepts in linguistics. These questions can only be answered by amassing further empirical evidence from various disciplines, especially from psycho- and neurolinguistics.

## Conclusion

Despite all controversies that still persist between minimalist and usage-based frameworks, there seems to be a broad agreement that there are “many mechanisms and pressures that shape the emergence of language” (MacWhinney, 2015, p. 12). There are many interesting parallels, especially between the complex adaptive system framework adopted in much research within usage-based and emergentist frameworks, on the one hand, and the evo-devo approach that has become influential in biolinguistics, especially in “biolinguistics 2.0”, on the other. What has become clear is that neither of the extreme positions sometimes found in the literature are wholly correct (Hurford, 2018) and that instead of making a distinct either/or decision, there is potential for the different approaches to find common ground on issues such as modularity, domain specificity vs. domain generality, innateness and development, and cultural and biological evolution.

Our view is that “progressive biolinguistics” (as represented in publications such as Di Sciullo and Boeckx, 2011; Boeckx and Benítez-Burraco, 2014a,b; Balari and Lorenzo, 2016, 2018; Boeckx and Martins, 2016; Boeckx, 2017) is partly converging with usage-based approaches. Traditional, or “orthodox” (Kirby, 2017; Balari and Lorenzo, 2018) biolinguistics, however, is not. This is evident, for example, in a recent paper by Crain et al. (2017), which compares the “biolinguistic approach” with the “usage-based approach” in child language acquisition. Crain et al. (2017) come to the conclusion that biolinguistic approaches are superior to usage-based approaches in terms of descriptive and explanatory adequacy. Yang et al. (2017) and Bolhuis (2019) represent further examples of the views held by “traditional” biolinguistics. Usage-based linguists, however, disagree with this assessment (see e.g., Ambridge and Lieven, 2011; Rowland, 2014; Ambridge, 2019). Here, we do in fact not see many points of convergence. This paper has therefore focused on the potential of convergence between certain strand of usage-based approaches and “progressive” biolinguistics.

Of course, the converse is also true. Not all versions of usage-based approaches are compatible or convergent with progressive biolinguistics. For example, Ambridge (2019) recently proposed a radical exemplar model of language acquisition that does not posit stored abstractions. Instead, novel forms are comprehended and produced *via* on-the-fly analogy across multiple stored exemplars. Clearly, again, there seems to be relatively little potential for points of convergence between these models and progressive biolinguistics. Overall, there are still many biolinguists who hold a more traditional view that is not compatible with the possible emerging consensus we have outlined here. Conversely, it is also true that not all proponents of usage-based approaches have fully integrated the perspectives from evo-devo, complex adaptive systems and dynamic system theory into their work. For example, Carpendale and Lewis (2015) criticize Tomasello (2014) for not adequately integrating the dynamic relationship of evolution and development as well as the interactive dimension of ontogeny into his model of the emergence of uniquely human cognition (see also Carpendale et al., 2018).^4^ Our comparison has therefore only scratched the surface of the conceptual convergences and divergences between the approaches. In particular, as illustrated in the discussion above, we have partly neglected the differences within the field of biolinguistics and usage-based approaches, respectively. As Balari and Lorenzo (2018) point out in their discussion of different ontological commitments regarding the status of language and the issue of modularity, “many middle ground positions exist that complicate the picture.” In addition, we have not discussed challenges that face both approaches equally, for example the question of how to integrate multimodality (Pleyer et al., 2017; Wacewicz and Zywiczynski, 2017) and embodiment (Ferretti et al., 2018; see also Gomez-Marin and Ghazanfar, 2019) into accounts of language evolution. The same holds for the challenges of integrating language evolution research with evo-devo research, a project that is still very much in its infancy (e.g., Benítez-Burraco and Boeckx, 2014). Overall, taking into account recent and future developments in (evolutionary) biology likely represents the most important step toward an integrative and biologically sound theory of language evolution. We also have not addressed the differences in the ontological conceptualization of language as an internal vs. external object, a topic that Balari and Lorenzo (2018) see as a fundamental axis of disagreement in the study of language.

Overall, though, it is an interesting perspective to see biolinguistics and usage-based and emergentist approaches being broadly compatible, which enables fruitful and structured debates about the mechanisms and pressures that exist on language emergence and their respective roles and interactions. Thus, we hope to have shown that the deep divide mentioned by Johansson (2014) is not as unbridgeable as it may seem and “there is actually much more complementarity than incompatibility between the findings and results of the two major research frameworks” (Mendívil-Giró, 2018).

## Author Contributions

All authors listed have made a substantial, direct and intellectual contribution to the work, and approved it for publication.

### Conflict of Interest

The authors declare that the research was conducted in the absence of any commercial or financial relationships that could be construed as a potential conflict of interest.

## Footnotes

^1^As one reviewer pointed out, it has to be noted that “species-specific” can actually mean different things in biolinguistics and usage-based approaches. For biolinguistics, this often means *Homo sapiens sensu stricto* (e.g., Berwick and Chomsky, 2016), which, for example, would exclude Neanderthals. Usage-based researchers, on the other hand, often see “species-specific” as referring to the human lineage while remaining open to the possibility that pre-hominins possessed a language-ready brain (cf., e.g., Dediu and Levinson, 2013, 2018; Johansson, 2015).

^2^For this as well as for the other points of convergence we attest in this paper it can of course be questioned whether they represent a positive development in the language sciences. Balari and Lorenzo (2018), for example, also see recent developments as paving the way to convergences between biolinguistics and “cognitive, externalist-inclined approaches” as represented, for example, by usage-based and Cognitive Linguistics (e.g., Croft and Cruse, 2004). However, they are much more skeptical whether this represents a positive development, as the convergences they observe “lead together (programmatically or not) to the dissolution of a well-delimited concept of language.”

^3^As one reviewer points out, research on the cultural evolution of the linguistic categorization of color serves as a paradigmatic example of fruitful attempts to explicate the biological foundations and socio-cultural factors influencing a given phenomenon (see e.g., Loreto et al., 2012; Gong et al., 2019).

^4^Overall, it has to be stated that at the moment, biolinguistics exhibits a much stronger commitment to integrating current trends and developments in biology than do most usage-based approaches. This then clearly presents a desideratum for usage-based approaches, which also stand to profit greatly from integrating biology more fully into their models of language.
